# Mitochondrial dysfunction, reactive oxygen species, and diabetes mellitus—A triangular relationship: A review

**DOI:** 10.17305/bb.2025.13145

**Published:** 2025-10-23

**Authors:** Mia Manojlovic, Sonja Zafirovic, Dragana Tomic Naglic, Edita Stokic, Manfredi Rizzo, Jasjit S Suri, Esma R Isenovic

**Affiliations:** 1 University of Novi Sad, Faculty of Medicine in Novi Sad, Novi Sad, Serbia; 2 Clinic for Endocrinology, Diabetes and Metabolic Disorders, University Clinical Center of Vojvodina, Novi Sad, Serbia; 3 Department of Radiobiology and Molecular Genetics, “VINČA” Institute of Nuclear Sciences – National Institute of the Republic of Serbia, University of Belgrade, Belgrade, Serbia; 4 School of Medicine, Promise Department, University of Palermo, Italy; 5 Ras Al Khaimah Medical and Health Sciences University, Ras Al Khaimah, United Arab Emirates; 6 Stroke Diagnostic and Monitoring Division, AtheroPoint, Roseville, CA, USA; 7 Department of Electrical and Computer Engineering, Idaho State University, Pocatello, ID, USA; 8 Global Biomedical Technologies, Inc., Roseville, CA, USA; 9 Symbiosis Institute of Technology, Nagpur Campus, Symbiosis International (Deemed University), Pune, India

**Keywords:** Oxidative stress, reactive oxygen species, mitochondrial dysfunction, diabetes mellitus, cardiovascular complications

## Abstract

Diabetes mellitus (DM) disrupts cellular homeostasis and is characterized by mitochondrial structural and functional impairments similar to those found in other metabolic disorders. Mitochondrial dysfunction (MD) leads to the excessive production of reactive oxygen species (ROS), which are central to the progression of cardiovascular (CV) disease—the leading cause of mortality associated with DM. ROS-driven oxidative stress (OS) is implicated in cardiac injury in both clinical and experimental contexts. This review synthesizes recent literature on the role of MD in the development and progression of DM and its associated CV complications, highlighting disrupted pathways that regulate the balance between ROS production and antioxidant defenses. We summarize alterations in mitochondrial dynamics—including fusion, fission, and mitophagy—mtDNA damage, and impaired oxidative phosphorylation characterized by dysregulated mitochondrial membrane potential (Δ Ψm), electron transport chain (ETC) defects, uncoupling, and substrate overload. Additionally, we discuss hyperglycemia-activated pathways such as polyol flux, AGE–RAGE interactions, protein kinase C/nicotinamide adenine dinucleotide phosphate (PKC/NADPH) oxidase activation, and poly (ADP-ribose) polymerase 1 (PARP-1)-mediated glyceraldehyde-3-phosphate dehydrogenase (GAPDH) inhibition, which contribute to inflammation, endothelial dysfunction, β-cell failure, insulin resistance, and micro/macrovascular injury. Diagnostic and biomarker strategies encompass mtDNA analysis, bioenergetic assays, metabolomics, proteomics, and imaging techniques including PET, MRI, and NIRS. Therapeutic approaches aimed at restoring mitochondrial function and mitigating OS include mitochondria-targeted antioxidants (such as MitoQ, CoQ10, SkQ1, SS-31, and Mito-TEMPO), metabolic drugs (including metformin and SGLT2 inhibitors), lifestyle modifications, and emerging gene-editing technologies. The interplay between mitochondria, ROS, and DM reflects a tightly regulated aspect of cellular physiology; while targeted and personalized strategies hold promise, they necessitate rigorous evaluation.

## Introduction

Diabetes mellitus (DM) is a chronic metabolic disorder characterized by persistent hyperglycemia due to impaired insulin secretion, action, or both [1]. As a highly prevalent condition, DM poses a significant public health challenge. The International Diabetes Federation (IDF) projects that by 2045, approximately 783 million adults, or one in every eight, will be living with diabetes [2]. Diabetes encompasses several forms, including type 1 and type 2 diabetes, gestational diabetes, monogenic diabetes, and prediabetes. Monogenic diabetes results from mutations in a single gene that affect insulin action or β-cell function. This category includes neonatal DM (NDM), mitochondrial diabetes, and maturity-onset diabetes of the young (MODY) [1].

Diabetes mellitus impacts cellular health and is characterized by mitochondrial structural and functional disruptions, which are also observed in other metabolic disorders. Mitochondrial dysfunction (MD) leads to the overproduction of reactive oxygen species (ROS), highly reactive molecules crucial for cell signaling and defense mechanisms. However, excessive ROS production results in oxidative stress (OS), adversely affecting cellular components [3]. MD, along with elevated ROS production, is regarded as a significant factor in the progression of cardiovascular (CV) pathology [4–7]. CV complications remain the leading cause of death among individuals with DM. Cardiac tissue exhibits a high density of mitochondria, as demonstrated in studies on conditions such as cardiac ischemia–reperfusion injury (IRI), diabetic cardiomyopathy, heart failure, and cardiac hypertrophy [8]. OS driven by ROS is considered a key factor in cardiac injury in both clinical and experimental models of diabetes. It is essential to recognize ROS as a notable instance of antagonistic pleiotropy. Under physiological conditions, ROS play a critical role in managing calcium signaling, triggering muscle contraction, facilitating cardiomyocyte development and maturation, and maintaining vascular tone. In contrast, dysregulation of ROS signaling under pathological conditions results in OS development [1, 4, 9].

## Search strategy

A comprehensive literature search was conducted in the PubMed and MEDLINE databases from October 1999 to September 2025. A combination of keywords and MeSH terms was utilized: “Mitochondrial Fusion and Fission,” “Mitochondrial Dynamics,” “Oxidative Stress,” “Reactive Oxygen Species,” “Mitochondrial Dysfunction and ROS,” “Diabetes Mellitus and Mitochondrial Dysfunction,” and “Mitochondrial Dysfunction and Cardiovascular Complications.” The search included review articles and original research articles, both in English and non-English articles containing English abstracts, as well as studies involving human and animal subjects.

## MD and OS in diabetes

### Mitochondrial dynamics: Fusion, fission, and mitophagy

A healthy population of mitochondria is crucial for cell survival. Mitochondrial fusion, fission, and trafficking are key processes that maintain mitochondrial morphology, function, and distribution, as these organelles are not static [1, 10]. Mitochondrial respiratory activity, mitochondrial DNA (mtDNA) distribution, cell survival, calcium signaling, and apoptosis are all highly dependent on fusion and fission processes [1, 10]. Several dynamin-related GTPases serve as master regulators of the balance between mitochondrial fusion (Mitofusin 1 [Mfn1], Mitofusin 2 [Mfn2], and Optic Atrophy 1 [OPA1]) and fission (dynamin-related protein 1 [Drp1]), thereby contributing to sophisticated mitochondrial dynamics [11, 12].

The integrity and functionality of mitochondria primarily hinge on fusion, which facilitates the exchange of mitochondrial contents. During mitochondrial fusion, mtDNA complementation allows normal mtDNA to compensate for damaged mtDNA, enabling mitochondria with defective mtDNA to survive [12]. Consequently, fusion acts as a guardian of genetic and biochemical homogeneity by diluting superoxide species and mutated DNA, alongside repolarizing membranes [10]. Impaired fusion and fragmented mitochondria compromise mitochondrial function. Conversely, fission is a division process that produces one or more daughter mitochondria, necessitating cytosolic Drp1 for execution [13]. During this process, damaged mtDNA is segregated from normal mtDNA, preserving portions of healthy mitochondria [12]. Excessive fission leads to mitochondrial fragmentation and mitophagy, selectively eliminating defective mitochondria. Thus, small individual mitochondria result from fission, while large interconnected mitochondrial networks arise from fusion. Insulin resistance (IR) and DM are classified as mitochondria-related diseases [1, 10].

Recent research implicates mitochondrial dynamics in regulating glucose metabolism and insulin signaling, contributing to the pathophysiology of obesity and type 2 diabetes. Mitochondria in pancreatic β cells are continuously recruited during fusion and fission processes. In nutrient-overloaded states, such as obesity and DM, mitochondrial fission is promoted, while fusion decreases, correlating with uncoupled respiration [10]. In contrast, genetic ablation of Drp1 in the liver (Drp1LiKO mice) results in decreased fat mass and lower HOMA-IR, thus protecting mice against high-fat diet-induced obesity and IR [14].

Diaz-Morales et al. posited that in type 2 DM (T2DM), poor glycemic control adversely affects mitochondrial dynamics, promoting leukocyte–endothelial interactions and facilitating the development of CV diseases. Their research demonstrated reduced mitochondrial fusion and enhanced fission in leukocytes from patients, with these characteristics becoming more pronounced in individuals with poor glycemic control [15].

Autophagy is a process that removes defective organelles by recycling essential components, with mitochondria undergoing a specialized form known as mitophagy [16]. Mitochondrial fission precedes mitophagy [1, 10]. As previously mentioned, during mitochondrial fission, mitochondria divide, allowing the damaged segment to be segregated from the healthy part for removal via mitophagy [12].

Phosphatase and tensin homolog (PTEN)-induced putative kinase 1 (PINK1), the ubiquitin ligase PARKIN, ubiquitin, and sequestosome-1 (p62/SQSTM1) are recognized as pivotal agents in the mitophagy process. PINK1 and PARKIN are indispensable for mitophagy [17]. Consequently, alterations in mitochondrial dynamics and mitophagy recycling through these proteins may create a conducive environment for the development of certain diseases [10].

### mtDNA damage and mutations in diabetic conditions

Mitochondrial DNA encodes essential components of the mitochondrial electron transport chain (ETC). Due to its close association with the ETC and the absence of protective histones, mtDNA is particularly vulnerable to oxidative damage. Elevated levels of ROS can lead to mtDNA impairment, resulting in base modifications, strand breaks, and deletions, ultimately disrupting mitochondrial protein synthesis and oxidative phosphorylation (OXPHOS) function [1, 18]. The accumulation of mtDNA mutations consequently impairs ETC activity, reduces ATP production, and increases ROS generation, creating a cycle of OS and dysfunctional mitochondria [19, 20].

Several factors can disrupt ATP synthesis through OXPHOS, including ETC activity, mitochondrial uncoupling, and substrate overload [21]. Specifically, ATP synthesis may be compromised due to impaired proton pumping across the inner mitochondrial membrane, as well as disruption of electron flow primarily resulting from dysfunctional ETC complexes I and III [22]. Furthermore, alterations in mitochondrial uncoupling proteins (UCPs—UCP1, UCP2, and UCP3), which are upregulated in DM, can compromise the regulation of mitochondrial membrane potential (Δ Ψm), further impairing ATP synthesis [23, 24]. Additionally, substrate overload due to excessive nutrient uptake poses a significant challenge to mitochondrial capacity, leading to elevated levels of fatty acids and glucose that ultimately result in defective substrate oxidation [25].

Given the crucial role of mtDNA integrity in metabolic homeostasis, a well-established correlation exists between mtDNA mutations, mitochondrial dynamics, DM, and its vascular complications [10]. The mutation m.8561 C>G in *MT-ATP6/8*, which encodes subunits of mitochondrial ATP synthase, has been associated with the onset of DM through diminished ATP production and assembly of mitochondrial ATP synthase [26]. Additionally, the m.A3243G mutation in the mitochondrial *tRNALeu* gene has been implicated in both DM and mitochondrial disease [27]. Notably, diabetes-related mutations and mitochondriopathies share numerous common mutations, suggesting a connection between mtDNA alterations and disease through similar pathways. To establish causality for each mutation, techniques such as cybrid or animal models must be employed for validation. Nonetheless, mtDNA analysis remains a significant tool for personalized management and the identification of at-risk individuals [1, 27].

## ROS in DM

### The impact of hyperglycemia on ROS production and the failure of antioxidant defense mechanisms in DM

**Figure 1.**
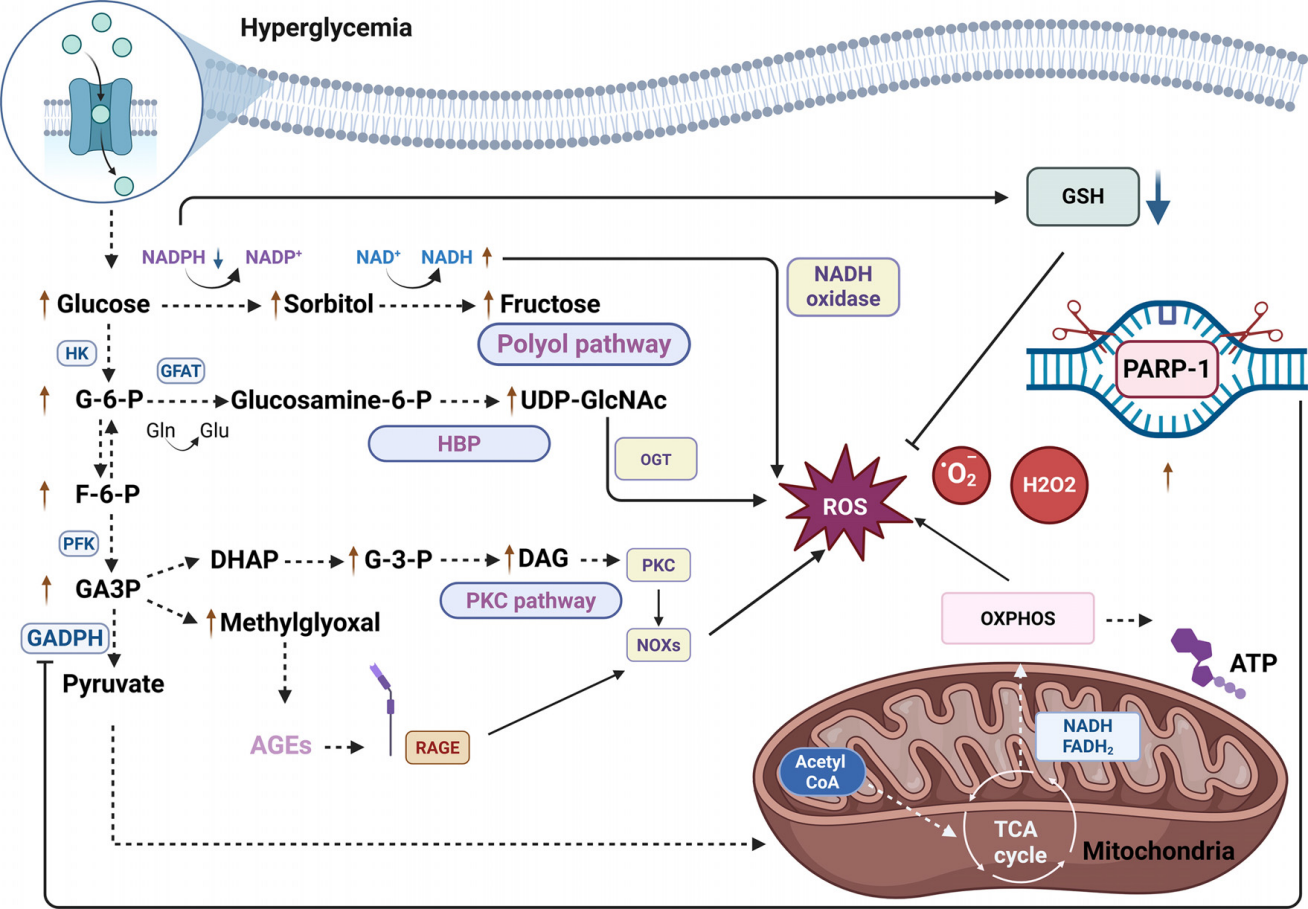
**Oxidative stress in diabetes mellitus.** In the state of hyperglycemia, the TCA cycle is fueled by increased mitochondrial pyruvate oxidation, thereby increasing mitochondrial ROS production. In parallel, several pro-oxidative pathways become activated, further amplifying the generation of ROS. These include the AGE and PKC pathways, driven by the accumulation of GA3P, as well as the hexosamine and polyol pathways, activated by hyperglycemia and elevated levels of F-6-P, respectively. Under conditions of hyperglycemia, hexokinase activity may be impaired, limiting the conversion of glucose to G-6-P. Consequently, excess glucose is shunted into the polyol pathway, resulting in substantial NADPH consumption. Since NADPH is essential for GSH regeneration, its depletion suppresses antioxidant defense systems and exacerbates oxidative stress. Within the hexosamine pathway, accumulation of UDP-GlcNAc promotes hyperactivation of O-GlcNAc transferase, leading to protein dysfunction and oxidative damage. Hyperglycemia also drives the activation of NOXs, thereby contributing to the overproduction of ROS. Excessive ROS accumulation causes DNA damage and activates DNA repair enzymes such as PARP-1. PARP-1, in turn, can inactivate GAPDH, leading to the buildup of metabolic intermediates, including GA3P, F-6-P, and G-6-P, which are prone to undergo diverse harmful reactions. Abbreviations: Acetyl-CoA: Acetyl coenzyme A; AGEs: Advanced glycation endproducts; ATP: Adenosine triphosphate; DAG: Diacylglycerol; DHAP: Dihydroxyacetone phosphate; F-6-P: Fructose 6-phosphate; FADH2: Reduced form of flavin adenine dinucleotide cofactor; G-3-P: Glycerol-3-phosphate; G-6-P: Glucose-6-phosphate; GA3P: Glyceraldehyde-3-phosphate; GAPDH: Glyceraldehyde-3-phosphate dehydrogenase; GFAT: Glutamine fructose-6-phosphate amidotransferase; Gln: Glutamine; Glu: Glutamic acid; GSH: Glutathione; HBP: Hexosamine Biosynthetic pathway; HK: Hexokinase; NAD+: Nicotinamide adenine dinucleotide; NADH: Reduced nicotinamide adenine dinucleotide; NADPH: Reduced nicotinamide adenine dinucleotide phosphate; NOXs: NADPH oxidases; OGT: O-linked N-acetylglucosamine transferase; OXPHOS: Oxidative phosphorylation; PFK: Phosphofructokinase; PKC: Protein kinase C; PARP-1: Poly [ADP-Ribose] polymerase-1; RAGEs: Receptor for advanced glycation endproducts; ROS: Reactive oxygen species; TCA: Tricarboxylic acid; UDP-GlcNAc: Uridine diphosphate N-acetylglucosamine.

Several mitochondrial pathways are altered in diabetes (Figure 1). Hyperglycemia can directly lead to increased ROS generation. Upon glucose entry into cells, two oxidation pathways may be activated, particularly the pentose phosphate and glycolytic pathways [4, 28]. Glycolysis is followed by the Krebs cycle, which generates nicotinamide adenine dinucleotide (NADH) and reduced flavin adenine dinucleotide (FADH2), which are subsequently utilized in OXPHOS to produce ATP. However, ROS byproducts, including hydrogen peroxide (H2O2), superoxide anions (O2●-), and hydroxyl radicals (●OH), are also produced during this process. Under normal physiological conditions, the antioxidant defense system, composed of enzymes such as superoxide dismutase (SOD), glutathione peroxidase (GPx), and catalase (CAT), effectively neutralizes these ROS [28–30].

In a hyperglycemic state, excessive ROS generation inhibits antioxidant systems, leading to DNA damage and activation of DNA repair enzymes, such as Poly (ADP-ribose) polymerase-1 (PARP-1) [4].

Subsequently, glyceraldehyde-3-phosphate dehydrogenase (GAPDH) can become inactivated by PARP-1, resulting in the accumulation of intermediates such as glyceraldehyde-3-phosphate (GA3P), fructose-6-phosphate (F-6-P), and glucose-6-phosphate (G-6-P), which are susceptible to diverse reactions that collectively contribute to OS (e.g., GA3P and G-6-P autooxidation, AGE precursor formation, and activation of protein kinase C (PKC) by GA3P) [31].

Significantly, in hyperglycemic conditions, hexokinase enzymatic activity may be impaired due to oversaturation, rendering the catalysis of G-6-P formation ineffective. Furthermore, glucose can enter the sorbitol pathway via aldose reductase, leading to the depletion of nicotinamide adenine dinucleotide phosphate (NADPH), which is normally a substrate for glutathione (GSH) production. This depletes antioxidant enzymes and exacerbates OS [28, 32].

Additionally, non-enzymatic reactions between glucose and proteins should not be overlooked, as they contribute to the formation of Amadori products, followed by advanced glycation end products (AGEs) that interact with AGE receptors (RAGE). This interaction induces OS and activates PKC, which enhances the upregulation of NADPH oxidase and lipoxygenase, further increasing ROS production [4, 33].

### ROS-mediated inflammation and diabetic complications

Inflammation and OS have a reciprocal relationship. In a state of general inflammation, ROS-producing macrophages become activated to eliminate pathogens. Concurrently, DM is characterized by persistent ROS generation, which depletes the antioxidant system and leads to cellular damage. In this metabolic disorder, pro-inflammatory cytokine expression is stimulated by both ROS and adipose tissue, including tumor necrosis factor-alpha (TNF-α) and interleukins 1 (IL-1) and 6 (IL-6) [34], thereby amplifying OS [35–37].

In the development of microvascular and macrovascular complications, hyperglycemia plays a critical role, as described in previous processes, triggering OS and continuous activation of the immune system, creating a vicious cycle [36]. Free radicals are integral to both the onset and progression of diabetic complications through pathways such as the aldose reductase pathway, the PKC pathway, and the production of AGEs. It is also important to note that the interplay between OS and inflammation can lead to increased secretion of monocyte chemoattractant protein-1 (MCP-1) and decreased levels of insulin-like growth factor-1, thereby promoting adipocyte differentiation and contributing to IR and hyperinsulinemia [36, 38].

In terms of complications, diabetic retinopathy is particularly concerning due to the high concentration of polyunsaturated fats in the retina, rendering it highly susceptible to OS [39]. In diabetic nephropathy, activation of NADPH oxidase with p47phox translocation drives ROS overproduction, reduces NO availability, and promotes proteinuria and glomerular matrix expansion. In one study, application of apocynin, an NADPH oxidase inhibitor, effectively prevented these changes [40]. Furthermore, OS may contribute to neuronal apoptosis and diminish regenerative capacity within the nervous system [41].

### OS-induced β-cell dysfunction and IR

Oxidative stress may deactivate the main signaling pathways essential for insulin actions (CB1, PI3K, and p38 MAPK). On the contrary, OS can also activate several stress-sensitive signaling pathways containing elements such as NF-κB, inducible nitric oxide (NO) synthase, and a class II histocompatibility complex, collectively leading to a great effect on insulin secretion and action. As a result, β-cells may change the shape, volume, and function of mitochondria, disrupting ATP-dependent K+ channels and impairing insulin secretion [36, 42].

Studies show that the liver’s expression levels of mitochondrial Mn-dependent *SOD2* and cytoplasmic Cu/Zn-dependent *SOD1* genes are below 50% of their maximum synthesis. In comparison, GPx and CAT levels are only about 5%. This makes islet cells highly susceptible to damage from ROS and other diabetogenic agents [42].

### The triangular interplay: MD, ROS, and diabetes

As previously noted, MD affects several key processes, including OXPHOS, ROS generation, and mtDNA integrity and dynamics. Collectively, these factors contribute to the manifestation of IR, β-cell dysfunction, and the onset of DM and its complications. Additionally, OS and T2DM are associated with alterations in mitochondrial membrane potential (Δ Ψm) in β-cells, potentially leading to pathological changes in mitochondrial dynamics that impair glucose-stimulated insulin secretion. This unravels a complex interplay of impaired fusion and fission processes, which are essential for mitochondrial lifespan [4, 10]. Notably, Δ Ψm serves as a central regulator of endothelial function in T2DM [43]. Hyperglycemia promotes Δ Ψm hyperpolarization, leading to diminished electron flux and increased ROS production via the ETC, as well as prolonging the half-life of ROS-generating intermediates [43]. Furthermore, studies in cultured cells demonstrate that excessive mitochondrial ROS production elevates the expression of endothelial adhesion molecules, reduces NO bioavailability, and increases pro-inflammatory cytokine levels. These changes occur as part of the inflammatory cascade, which is partially mediated through the activation of NF-κB and protein kinase C-β [43, 44]. Moreover, findings from a study in human subjects conducted by Kizhakekuttu et al. [45] underscore the importance of Δ ψm as a determinant that, at least partially through mitochondrial ROS generation, likely modulates the endothelial phenotype as well as vascular endothelial function in individuals with T2DM, notably through rapid adjustments in arteriolar endothelial responsiveness.

## Diagnostic tools and biomarker insights

While the clinical framework for diagnosing diabetes is well established, insight into the role of MD in its onset remains limited and underdeveloped [1]. From the perspective of adjunct diagnostic modalities and biomarker utilization, several approaches have been proposed, including mtDNA analysis, respiratory chain enzyme assays, advanced imaging techniques, as well as metabolomic and proteomic profiling [21].

For instance, mtDNA analysis may serve as an early warning tool to identify individuals at heightened risk of developing diabetes, thus enhancing the reach of personalized medicine. This analysis encompasses the evaluation of mutations, copy number variations, and overall genomic integrity [20]. Respiratory chain enzyme assays facilitate the assessment of individual ETC complexes and can be conducted using tissue biopsies or cell cultures derived from diabetic patients [46].

Metabolomic profiling, which involves the analysis of small-molecule metabolites in biological samples, identifies metabolic signatures associated with β-cell dysfunction, IR, OS pathways, and diabetes-related complications. This approach provides valuable insights into systemic metabolic abnormalities and mitochondrial impairment [47].

**Figure 2.**
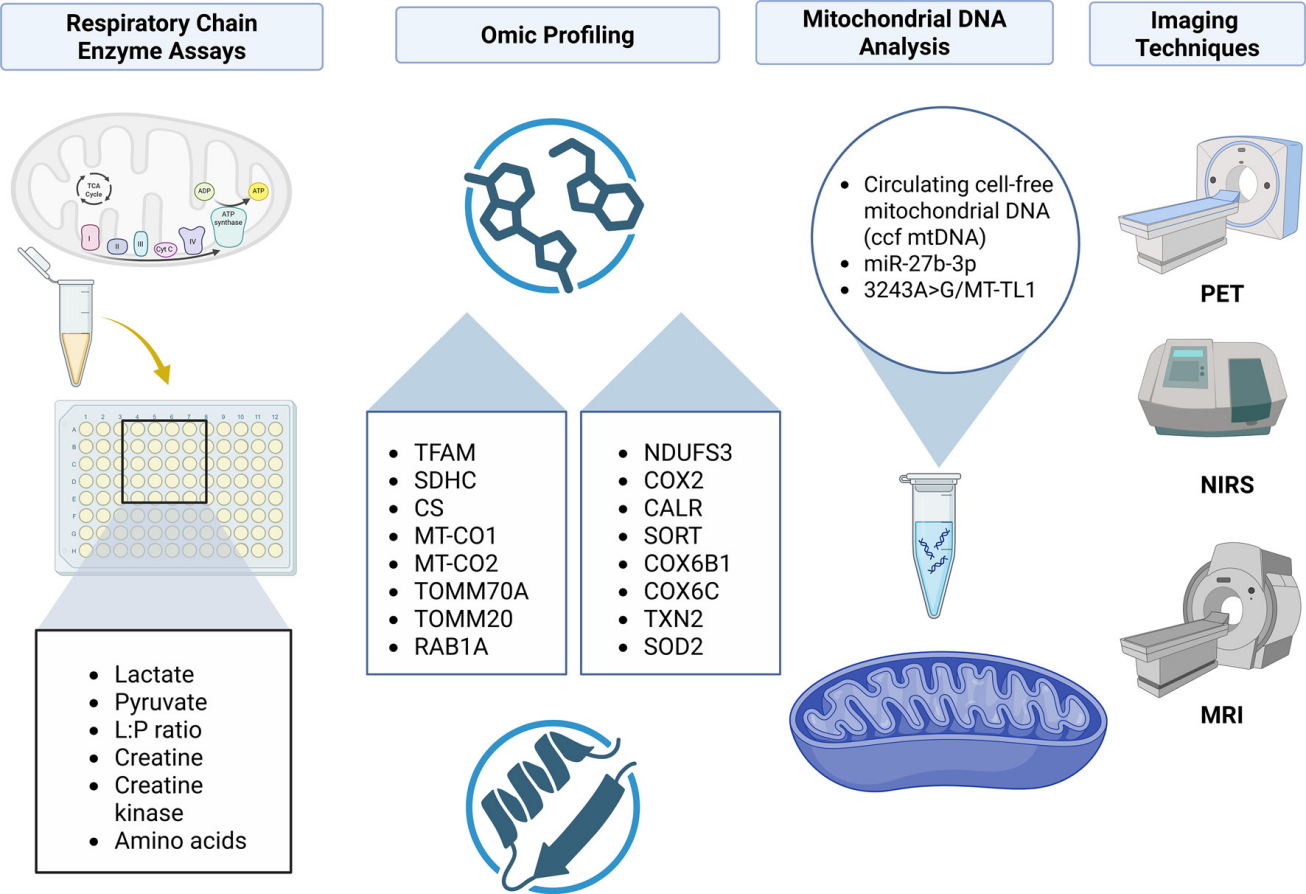
**Diagnostic tools and biomarkers of mitochondrial dysfunction.** Overview of adjunct approaches to assess mitochondrial dysfunction in diabetes: Enzyme assays with bioenergetic markers, omics profiling, mitochondrial DNA markers, and noninvasive imaging (PET, NIRS, MRI). Abbreviations: L:P ratio: Lactate/pyruvate ratio; PET: Positron emission tomography; NIRS: Near-infrared spectroscopy; MRI: Magnetic resonance imaging; mtDNA: Mitochondrial DNA; TFAM: Transcription factor A, mitochondrial; SDHC: Succinate dehydrogenase complex subunit C; CS: Citrate synthase; MT-CO1/2: Mitochondrially encoded cytochrome c oxidase subunits 1/2; TOMM70A/TOMM20: Translocase of outer mitochondrial membrane 70/20; RAB1A: Ras-related protein Rab-1A; NDUFS3: NADH dehydrogenase (ubiquinone) iron–sulfur protein 3; COX2: Cytochrome c oxidase subunit 2 (MT-CO2); COX6B1/6C: Cytochrome c oxidase subunits 6B1/6C; CALR: Calreticulin; SORT: Sortilin; TXN2: Thioredoxin 2; SOD2: Superoxide dismutase 2; miR-27b-3p: MicroRNA-27b-3p; MT-TL1: Mitochondrially encoded tRNA-Leu(UUR); m.3243A>G: A-to-G variant at mtDNA position 3243 in MT-TL1.

Non-invasive imaging techniques, such as positron emission tomography (PET), magnetic resonance imaging (MRI), and near-infrared spectroscopy (NIRS), offer opportunities to assess tissue metabolism and mitochondrial function *in vivo*. Collectively, these strategies highlight the urgent need to define and classify prognostic biomarkers in diabetic patients at risk of MD, ultimately aiming to develop tailored therapeutic and preventive interventions [48]. Figure 2 presents a summary of the discussed diagnostic tools and potential biomarkers of MD.

## Therapeutic strategies targeting mitochondria and ROS in diabetes

### Mitochondria-targeted antioxidants, pharmacological interventions, and nutritional and lifestyle approaches

When considering mitochondria as therapeutic targets, it is essential to acknowledge their extraordinary complexity as central integrators of oxidative metabolism, cellular signaling, and apoptotic pathways. This complexity poses challenges in targeting mitochondria, as evidenced by inconsistent results from preclinical and sometimes clinical trials involving mitochondria-directed antioxidants or peptides. Nonetheless, therapeutic benefits have been observed in certain diabetes-related complications, including impaired wound healing, diabetic nephropathy, diabetic neuropathy, and hepatic steatosis. Consequently, mitochondria represent a promising area for scientific exploration in developing novel targeted therapies. Advances in technology, such as molecular dynamics simulations and molecular docking, may enable the development of interventions aimed at specific aspects of mitochondrial biology, including mitochondrial dynamics, mitophagy, ionic overload, and the regulation of mitochondrial channels, such as uncoupling proteins (UCPs) [49].

**Figure 3.**
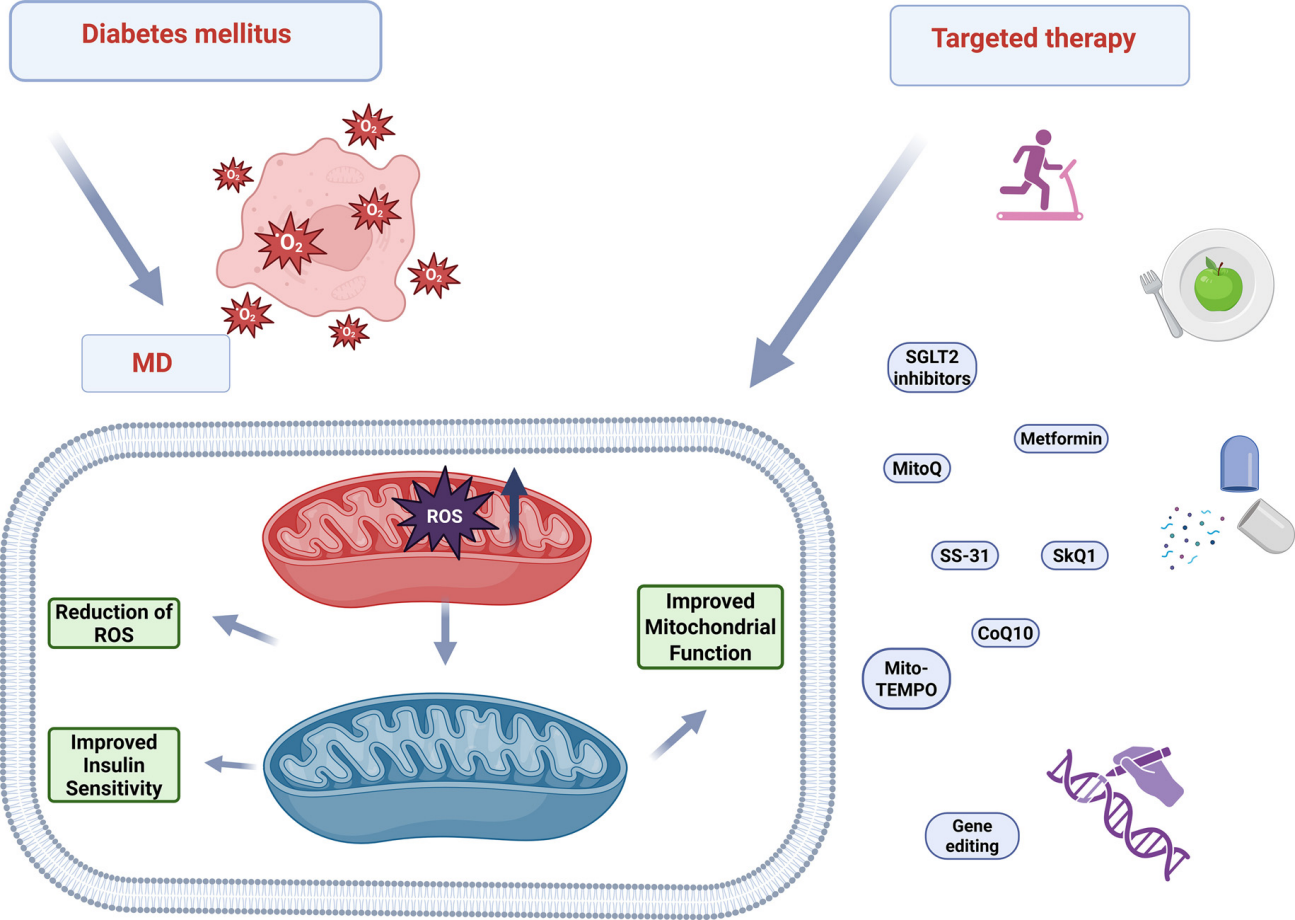
**Possible therapeutic strategies targeting mitochondria and ROS in diabetes.** Mitochondria-targeted antioxidants (MitoQ, CoQ10, SkQ1, SS-31, Mito-TEMPO), pharmacologic modulators (metformin, SGLT2 inhibitors), lifestyle measures (exercise, diet), and emerging gene editing converge to reduce ROS, restore mitochondrial function, and improve insulin sensitivity. Abbreviations: DM: Diabetes mellitus; MD: Mitochondrial dysfunction; ROS: Reactive oxygen species; MitoQ: Mitoquinone; CoQ10: Coenzyme Q10; SkQ1: Plastoquinonyl decyltriphenylphosphonium; SS-31: Elamipretide; Mito-TEMPO: Mitochondria-targeted TEMPO; SGLT2 inhibitors: Sodium–glucose cotransporter 2 inhibitors.

Studies indicate that traditional antioxidants, such as vitamins C and E, do not effectively address diseases involving oxidative damage to mitochondria, likely due to the limited amount of these antioxidants reaching the mitochondria, with the remainder distributed throughout the body. As a result, the identification of antioxidants that can directly target mitochondria has become necessary [50]. Possible therapeutic strategies targeting mitochondria and ROS in diabetes are summarized in Figure 3.

MitoQ is a mitochondria-targeted derivative of coenzyme Q10 and is among the most extensively studied antioxidants. Its unique structure, featuring a ubiquinone moiety linked to a triphenylphosphonium moiety, facilitates MitoQ’s transfer and accumulation in mitochondria [51]. However, the effects of MitoQ on glycemic control in preclinical models of diabetes and obesity have been inconsistent. For instance, in ATM+/ /ApoE /  mice, which develop metabolic syndrome rapidly on a high-fat diet, a 7-week treatment with MitoQ improved glucose tolerance and reduced fasting glucose, insulin, triglycerides, and cholesterol levels [52]. Similarly, MitoQ enhanced insulin secretion in pancreatic β cells exposed to hyperglycemic conditions, mimicking human hyperglycemia [53]. In contrast, two recent studies reported that MitoQ did not lower glycemia in rat models of type 2 diabetes induced by a high-fat diet and streptozotocin. This discrepancy may be attributed to the severity of diabetes in these models, which is greater than that typically observed in humans [54, 55]. Comparable findings were noted in a type 1 diabetes model using Akita (Ins2+/ AkitaJ) mice [56]. Despite these limitations, the therapeutic potential of MitoQ should not be overlooked, as promising benefits have been consistently observed in diabetic complications, including kidney injury [56, 57], neuropathy [55], and hepatic steatosis [52, 54]. It was shown that MitoQ improved microvascular function in patients with chronic kidney disease, partially by reducing the NADPH oxidase [58]. Also, treatment of T2DM patients with MitoQ decreased mitochondrial ROS production, as well as the level of NFκB-p65 and TNFα, supporting the idea that MitoQ shows anti-inflammatory and antioxidant properties [59].

Coenzyme Q10 protects cells and mitochondria from oxidative damage, decreases ROS generation, and enhances antioxidant defenses. CoQ10 reduces electron leakage in mitochondrial Complex II, facilitating electron transfer to Complex III and thereby indirectly decreasing superoxide production in hyperglycemic conditions. This contributes to the protection of endothelial cells and fosters favorable oxidative conditions in the cell [4, 60, 61]. Diabetic patients receiving 150 mg of CoQ10 for 12 weeks exhibited decreased levels of triglycerides, HDL-C, fasting plasma glucose, and hemoglobin A1C, although LDL-C levels increased [62]. Similarly, diabetic patients with neuropathic signs who received 200 mg/day of CoQ10 showed improved insulin sensitivity and total antioxidant capacity (TAC), alongside a reduction in high-sensitivity C-reactive protein (hsCRP) levels [63].

Furthermore, vigorous antioxidant properties aimed at mitochondrial bioenergetics are attributed to other mitochondrial-targeted antioxidants, such as SkQ1 and SS-31 (elamipretide), making them promising adjunctive therapies for DM [64]. SkQ1 improves the functioning of mitochondria like a MitoQ, while SS-31 affects MD by improving fusion, reducing OS damage, and IR [51]. Regarding SkQ1, similar mixed results have been reported in the context of hyperglycemia and OS. For instance, in a study using db/db mice, a model of T2DM, administration of SkQ1 for up to 12 weeks was ineffective in reducing HbA1c or blood glucose levels, but decreased the level of lipid peroxidation end products [65]. Conversely, in rats with alloxan-induced type 1 diabetes, pre-treatment with SkQ1 inhibited diabetes onset, likely due to its antioxidant properties, as alloxan induces diabetes by causing oxidative damage to pancreatic β cells [66, 67]. To address these translational gaps, it has been proposed that SkQ1 should be evaluated in models that more closely resemble human diabetes or in established alloxan-induced diabetes [49]. Nevertheless, when considering diabetes-related complications, SkQ1 appears to exert protective effects, most notably by promoting wound healing, despite the absence of glucose-lowering action [68]. Preclinical, *in vivo* and *in vitro* studies show that treatment with SS-31, a novel mitochondria-targeting antioxidant, ameliorates high glucose-induced MD and myocardial injury [69]. In patients with T2DM, SS-31 treatment decreased mitochondrial and total ROS, as well as the level of indicators of inflammation, NFκB-p65, and TNFα. Also, mitochondrial function was restored, most likely by increasing the level of SIRT1 [70].

Mito-TEMPO is a mitochondria-targeted SOD mimetic that neutralizes free radicals via conjugation of piperidine nitroxide with a triphenylphosphonium group. By converting superoxide anions into oxygen and hydrogen peroxide, it alleviates mitochondrial OS, a key factor in diabetic complications [71]. Beyond reducing ROS, apoptosis, and hypertension, Mito-TEMPO enhances endothelial function, restores mitochondrial complex II activity diminished by IR, and modulates key signaling pathways, including ERK1/2 and GLP-1/CREB/adiponectin [72]. In diabetic mice, Mito-TEMPO alleviated myocardial dysfunction and decreased mitochondrial ROS [71], while in diabetic nephropathy models of T1D and T2D inhibited the PKR/eIF2α pathway and reduced mitochondrial ROS [73].

In the context of human studies on mitochondria-targeted antioxidants, a recent systematic review and meta-analysis of randomized controlled trials evaluated nineteen studies (*n* ═ 884 participants) investigating agents such as elamipretide, MitoQ, and MitoTEMPO. The analysis concluded that although short-term interventions suggest these compounds are generally well tolerated, there is currently insufficient evidence from RCTs to support their efficacy in improving glycemic control. Future research should focus on assessing mitochondria-targeted antioxidants in specific patient populations and under conditions of hyperglycemia [64]. The results of animal and human studies are summarized in Table 1.

Besides antioxidants, various metabolic modulators that enhance mitochondrial function and cellular metabolism exhibit promising therapeutic properties. For instance, metformin, a widely used medication for diabetes, significantly influences mitochondrial dynamics. Its therapeutic effects primarily arise from the inhibition of mitochondrial complex I activity and the reduction of OS-induced damage [74]. Metformin plays a vital role in maintaining cellular health by promoting mitochondrial autophagy and facilitating the removal of dysfunctional mitochondria [74, 75].

**Table 1:** Mitochondria-targeted antioxidants: Animal and human studies

**Treatment/antioxidant**	**Condition**	**Model**	**Key outcome**	**Ref**
MitoQ	Atherosclerosis metabolic syndrome	Preclinical/*in vivo* ApoE( / ) mice ATM(+/ )/ApoE( / ) mice	Improved glucose tolerance ↓ Fasting glucose, ↓ Insulin ↓ Triglycerides, ↓Cholesterol	[52]
	Hyperglycaemia	Preclinical/*in vitro* pancreatic β cell line INS-1E	↑Insulin secretion ↓ GSH levels ↓ER stress markers (GRP78, P-eIF2α) ↓ NFκB-p65	[53]
	T2D	Preclinical/*in vivo* rats (high-fat diet+streptozotocin)	↓ Liver fat ↓Hydroperoxside Unchanged glycemia	[54]
	Pre-diabetes late-stage T2D	Preclinical/*in vivo* rats (high-fat diet+streptozotocin)	Unchanged glycemia	[55]
	T1D diabetic nephropathy	Preclinical/*in vivo* Ins2(+/)–(AkitaJ) mouse model (Akitamice)	Improved tubular function Improved glomerular function ↓Urinary albumin	[56]
	Diabetic kidney disease	Preclinical/*in vivo* diabetic db/db mice	↑OCR ↓ATP	[57]
	T2D	Human/*ex vivo* leukocytes from T2D patients	↓ROS ↓NFκB-p65 ↓TNFα	[59]
	Chronic kidney disease	Human/pilot study	Improved vascular function ↓NADPH oxidase	[58]
CoQ10	Diabetes	Human/randomized, double blind, placebo-controlled trial	↓FPG ↓HbA1C ↓Triglyceride ↓ HDL-C ↑LDL-C	[62]
	Diabetes neuropathic signs	Human/randomized placebo-controlled clinical trial	↑Insulin sensitivity ↑ TAC ↓CRP	[63]
SkQ1	T2D	Preclinical/*in vivo* C57BL/KsJ-db-/db- mice	↓TBARS Unchanged hyperglycemia Improved healing of skin wounds	[65]
	T1D	Preclinical/*in vivo* rats (pre-therapy SkQ1+ alloxan)	Normalized Blood Glucose Level	[66]
SS-31	Diabetic cardiomyopathy	Preclinical/*in vivo; in vitro* diabeticC57 BL/6J mice; H9C2 cells	Ameliorates mitochondrial dysfunction and myocardial injury	[69]
	T2D	Human/*ex vivo* leukocytes from T2D patients	↓NFκB-p65 ↓TNFα ↑SIRT1 ↓total ROS ↓Mitochondrial ROS	[70]
mitoTEMPO	T2D T1D	Preclinical/*in vivo* streptozotocin and db/db mice	↓Mitochondrial ROS Alleviated myocardial dysfunction	[71]
	Diabetic nephropathy T2D T1D	Preclinical/*in vivo; in vitro* streptozotocin-and db/db mice; HK-2 cells	↓Mitochondrial ROS ↓PKR/eIF2α	[73]

Abbreviations: CoQ10: Coenzyme Q10; CRP: C-reactive protein; FPG: Fasting plasma glucose; GSH: Glutathione; HbA1C: Hemoglobin; HDL-C: High-density lipoprotein cholesterol; LDL-C: Low-density lipoprotein cholesterol; NADPH: Nicotinamide adenine dinucleotide phosphate; NF-κB: Nuclear factor kappa B p65 subunit; OCR: Mitochondrial oxygen consumption; ROS: Reactive oxygen species; SIRT1: Class III histone deacetylase sirtuin-1; TAC: Total antioxidant capacity; TBARS: Thiobarbituric acid-reactive substances; T1D: Type 1 diabetes; T2D: Type 2 diabetes; TNF-α: Tumor necrosis factor-alpha; ↓: Decrease; ↑: Increase.

Sodium-glucose cotransporter 2 inhibitors are effective therapeutic agents that function by blocking the renal reabsorption of glucose, which increases its urinary excretion and leads to glucosuria. This primary mechanism is associated with downstream metabolic effects, including a shift toward ketogenesis, a reduction in inflammation, and an enhancement of mitochondrial function [76]. Consequently, their beneficial effects on improving mitochondrial function and mitigating OS, which are crucial for their cardiorenal protective effects, should not be overlooked [77].

From the perspective of lifestyle interventions, including dietary modifications and physical activity, the primary mechanism focuses on modulating energy balance and substrate utilization. Various nutritional patterns that promote overall metabolic health, such as ketogenic, low-carbohydrate, and intermittent fasting regimens, have gained significant attention in recent years [1]. These approaches enhance mitochondrial biogenesis and improve insulin sensitivity. Additionally, specific nutrients and bioactive compounds, including 5-aminoimidazole-4-carboxamide ribonucleotide (AICAR), GW501516, and epicatechin, have been investigated for their ability to activate key metabolic regulators such as AMP-activated protein kinase (AMPK) and peroxisome proliferator-activated receptors (PPARs) [78]. Furthermore, it is well established that physical activity significantly benefits mitochondrial function and insulin sensitivity while simultaneously reducing OS [1].

### Emerging therapies: Gene editing

Gene expression and mtDNA integrity represent important targets for future gene-based therapies aimed at treating MD in DM and CV conditions [79]. Current treatments for DM-related mtDNA mutations primarily address symptoms; thus, emerging mitochondrial gene therapies aim to rectify mtDNA mutations and restore mitochondrial function [80]. Although mitochondrial gene therapy is a relatively recent concept, significant progress has been made, yet many challenges remain. The multicopy nature of the mitochondrial genome complicates the diagnosis and prediction of mtDNA disease progression. While heteroplasmy manipulation has been a research focus for decades, effective and practical methods have only recently begun to materialize. Mitochondrial gene editing technologies are being developed to specifically target variant mtDNA molecules, thereby steering a heteroplasmic state toward a healthier, wild-type mtDNA population [81].

Current mitochondrial editing techniques are based on two primary strategies. The first aims to remove mutated mtDNA using mitochondrial-targeted nucleases, while the second seeks to modify mtDNA through mitochondrial-targeted base editors. Mitochondrial-targeted nucleases, such as transcription activator-like effector nucleases (TALENs) and zinc-finger nucleases (ZNFs), provide a CRISPR-free alternative capable of cleaving double-stranded mutant mtDNA within mitochondria. However, these editing tools face significant limitations, including susceptibility to nonspecific DNA interactions and the need for extensive engineering for each new target site, which affects their clinical applicability. Since mitoTALENs and mitoZFN can only remove mutated mtDNA without directly repairing specific mutations, base editors have been developed. Mitochondrial base editing technology enables nucleotide conversions, most frequently from C to T or A to G, facilitating the repair of mutated mtDNA and promoting a healthy mtDNA population. Overall, the further development of more precise editors and improved delivery systems is essential for transitioning these technologies from experimental to clinical application [82–85].

While current advancements in mitochondrial gene editing primarily rely on allotopic expression and DNA-editing enzymes/base editors designed for mitochondrial function, conventional CRISPR delivery to mtDNA remains a significant hurdle, despite the widespread use of the CRISPR-Cas9 system as a genome editing tool [1, 86, 87]. Allotopic expression entails the nuclear relocation of mitochondrial genes to bypass mtDNA mutations and restore mitochondrial protein synthesis. The application of CRISPR-Cas9 for mtDNA editing continues to be debated, primarily due to its inefficiency, despite its potential to target mitochondria. These challenges will shape future research directions, which may focus on enhancing mitochondrial transport mechanisms to improve the delivery efficiency of editing components into mitochondria, as well as developing Cas protein variants with higher editing efficiency through genetic engineering [80–82].

## Conclusion

This review critically examines recent literature on the role of MD in the development and progression of DM and its associated CV complications. It also provides an overview of the disrupted molecular pathways related to the balance of ROS production and antioxidant defense in this pathology. Furthermore, the review explores the latest strategies for restoring mitochondrial function and reducing OS, highlighting recent advancements in targeted treatments and lifestyle interventions. The precise regulation of cellular physiology underpins the interaction between mitochondria, ROS, and DM. The heterogeneity of DM phenotypes underscores the importance of personalized medicine approaches tailored to specific disease manifestations.
